# Accurate Orientation Estimation Using AHRS under Conditions of Magnetic Distortion

**DOI:** 10.3390/s141120008

**Published:** 2014-10-24

**Authors:** Nagesh Yadav, Chris Bleakley

**Affiliations:** Complex and Adaptive Systems Laboratory, School of Computer Science and Informatics, University College Dublin, Dublin 4, Ireland; E-Mail: chris.bleakley@ucd.ie

**Keywords:** AHRS, particle filter, data fusion, inertial measurement units, magnetic distortion, motion capture

## Abstract

Low cost, compact attitude heading reference systems (AHRS) are now being used to track human body movements in indoor environments by estimation of the 3D orientation of body segments. In many of these systems, heading estimation is achieved by monitoring the strength of the Earth's magnetic field. However, the Earth's magnetic field can be locally distorted due to the proximity of ferrous and/or magnetic objects. Herein, we propose a novel method for accurate 3D orientation estimation using an AHRS, comprised of an accelerometer, gyroscope and magnetometer, under conditions of magnetic field distortion. The system performs online detection and compensation for magnetic disturbances, due to, for example, the presence of ferrous objects. The magnetic distortions are detected by exploiting variations in magnetic dip angle, relative to the gravity vector, and in magnetic strength. We investigate and show the advantages of using both magnetic strength and magnetic dip angle for detecting the presence of magnetic distortions. The correction method is based on a particle filter, which performs the correction using an adaptive cost function and by adapting the variance during particle resampling, so as to place more emphasis on the results of dead reckoning of the gyroscope measurements and less on the magnetometer readings. The proposed method was tested in an indoor environment in the presence of various magnetic distortions and under various accelerations (up to 3 *g*). In the experiments, the proposed algorithm achieves <2° static peak-to-peak error and <5° dynamic peak-to-peak error, significantly outperforming previous methods.

## 1. Introduction

Attitude heading reference systems (AHRS) based on MEMS (microelectromechanical systems) technology are becoming popular for applications, such as underwater navigation, aircraft guidance control and motion capture (Mocap) [1–3]. Typically, an AHRS consists of a triaxial accelerometer, triaxial gyroscope and triaxial magnetometer. In mocap applications, multiple AHRS are affixed to the human body and are used to monitor the orientation of each section of the body. Knowledge of the human body joint configuration is then used to derive the full body kinematics. The conventional algorithm for orientation estimation involves using estimates of the Earth's gravity vector and the Earth's magnetic field reference. Naturally, the horizontal component of the Earth's ambient magnetic field originates from the magnetic south pole and points towards the magnetic North pole. Magnetic field strength measurements, relative to previously measured calibration values, from the magnetometer are used to calculate the heading of the AHRS. The direction of gravity is calculated using the accelerometer readings. However, under external magnetic field disturbances, for example when the AHRS is close to a ferrous or magnetic object, the estimated heading is incorrect, and so, the orientation estimates are inaccurate. Thus, it is desirable to devise a means of mitigating magnetic disturbances.

In this paper, the authors propose a novel orientation estimation algorithm that is robust to magnetic disturbances and performs well under a range of velocities and accelerations. Magnetic distortions are detected by using magnetic strength in conjunction with magnetic dip angle. A particle filter is used for orientation estimation. Particles are sampled from the state space based on a variance parameter that is dependent on the presence of magnetic distortion. The cost function for evaluating each particle in the state space of the particle filter is adaptive. When distortion is detected, adaptation reduces the reliance of the estimation on the magnetometer readings and places more emphasis on the results of dead reckoning based on the gyroscope readings. In the quasistatic condition, inclination estimates derived from the accelerometer are used to evaluate orientation particles. Whereas, under fast motion, the magnetic dip angle derived from the magnetometer and gyroscope is used for evaluating each orientation particle.

The key novel aspect of the proposed algorithm is the use of the magnetic dip angle, in conjunction with the gravity vector and calibrated magnetic field strength, to detect magnetic field distortion prior to mitigation. The use of derived dip angle significantly increases the sensitivity of the detection algorithm. In addition to detecting proximity to a ferrous or magnetic object, the proposed method also detects the presence of a distant ferrous or magnetic material, which alters the direction of the magnetic field, but remains largely undetected using previous methods. The conventional autoregressive model for modeling magnetic distortions is incapable of efficiently correcting magnetic distortion in cases where the object enters the distorted area at low speed (less than 0.2 m/s for a system providing 50-Hz orientation updates). This is due to the inability of the method to detect magnetic distortion at boundary regions of the magnetically distorted area. A perceptible change in magnetic strength (*i.e.*, a change in magnetic strength that is above the noise floor) is observed only after the magnetometer is close to the distorting object. This is further demonstrated by experiments in Section 3.1. An adaptive cost function enables the correction to be performed under noisy conditions. The proposed method is equally applicable at all speeds and shows an overall improvement in orientation estimation under high acceleration.

The remainder of this paper is organized as follows. Section 2 presents related work in the area of orientation estimation using inertial sensors and magnetometers and related methods for distortion mitigation. Section 3 introduces the proposed method. Section 4 presents the experimental results and discussion. Section 5 presents the conclusions and future work.

## 2. Related Work

A typical inertial motion capture system uses an accelerometer, gyroscope and magnetometer to estimate 3D orientation. The accelerometer and gyroscope measure the applied accelerations and angular velocities, while the magnetometer readings are used to determine the heading of the device. Two types of attitude heading reference systems (AHRSs) have been studied.

The active type uses a magnetic field generator. The induced magnetic field strength in the receiver coils is proportional to the distance from the source [4]. This is used to estimate the range of the receiver from the active magnetic source. For mitigating magnetic disturbances near a fixed distortion source, the authors propose the use of a lookup table that stores the additive vectors and rotations associated with the 3D position. The difficulty with using an active magnetic tracker is that the magnetic dipole strength decreases as the distance between the source and receiver increases [5]. Hence, generators are either mains powered and bulky or short range.

The second category of AHRS uses the Earth's ambient magnetic field to estimate the heading of the AHRS. Magnetic distortions of the Earth's magnetic field are investigated in [6]. The authors conclude that both the magnetic dip angle and heading estimates are inaccurate in perturbed magnetic fields. The authors suggest performing mapping of the ferromagnetic characteristics of a room before performing motion capture. The difficulty with this approach is that when only using an AHRS, position is unknown. Thus, correction based on a 3D map is impractical. The authors also conclude that the metallic structure of buildings and the presence of ferrous materials cause magnetic distortion, and it is desirable to keep the magnetometer at least 40 cm above the floor to reduce distortion effects.

The problem of the detection and correction of magnetic disturbances has been examined by a number of authors. Magnetic field strength was used in [7] for detecting the presence of magnetic distortions. Magnetic dip angle was used for modeling the magnetic distortion as an autoregressive process. A Kalman filter was used to estimate the heading and attitude of the AHRS. However, the authors report a problem detecting distortions in cases when an object enters the erroneous area at low speeds (less than 0.2 m/s for a system providing 50-Hz orientation updates). This is due to the inability of the method to detect magnetic distortion at boundary regions of the magnetically distorted area. A perceptible change in magnetic strength (*i.e.*, a change in magnetic strength that is above the noise floor) is observed only after the magnetometer is close to the distorting object. This is further demonstrated by experiments in Section 3.1. The autoregressive model fails to detect small variations (*i.e.*, close to the noise floor of the sensor) in magnetic strength due to a distant ferrous object. Commercially available AHRS-based products, such as MTi, MTxfrom Xsens, use a magnetic field mapper to perform local soft iron and hard iron calibrations [8]. Again, this approach fails if the position is unknown. A method for characterizing magnetic distortions internal to a vehicle was proposed in [9]. Magnetic distortions were characterized by using external magnetic field measurements, internal measurements and the known orientation of a vehicle. This method is not applicable for an indoor motion capture scenario, mainly because of varying arrangements of magnetic materials within rooms. A Kalman filter-based algorithm was used with reject and reset phases for accurate flight dynamics in [10]. A reset phase was used to reset the filter errors during quasirectilinear uniform motion. Errors in the magnetometer readings were calibrated using a non-linear integrated filter model. For large initial heading errors (more than 2°), a particle filter was shown to outperform the extended Kalman filter in [11]. A gradient descent algorithm was used for magnetic distortion compensation and orientation estimation [12]. Gyroscope measurement error was derived as a quaternion derivative using accelerometer and magnetometer data. The main disadvantage of using a gradient descent algorithm is the responsiveness of the estimation algorithm. The estimator ignores sharp changes in the magnetic field, and thus, short-term magnetic distortions are undetected and remain uncompensated.

**Figure 1.**
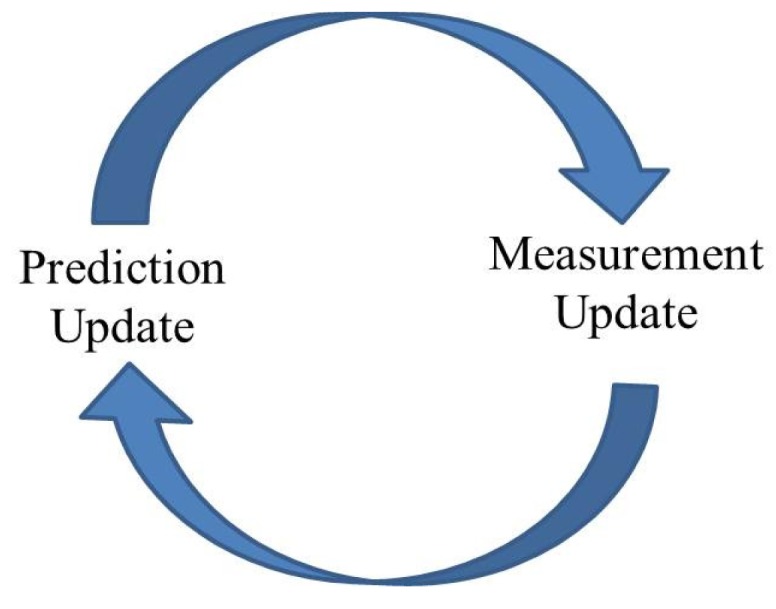
Generic Kalman filter propagation states [13].

A Kalman filter is a statistical, recursive estimation technique using noisy measurements [13]. The Kalman filter is an approach that is used to to estimate the state of a discrete-time controlled process. The Kalman filter uses a conventional iterative prediction/correction approach, as shown in Figure 1. Consider a system that is governed by the linear stochastic difference in Equation (1):

(1) $$X_{t+1}=AX_{t}+Bu_{t}+w_{t}$$

*X_t_* is the state vector consisting of variables that define the current state of the system. *A* is the state transition matrix; *u_t_* is the input to the system at time instant *t*; and *w_t_* is the process noise. This forms the basis of state prediction based on the current input or the motion model. This estimated state is also known as an *a priori* estimate.

The measurement vector *Z_t_* is derived from the *a priori* estimate using the measurement matrix *H*:

(2) $$Z_{t}=HX_{t}+\upsilon_{k}$$

where *υ_k_* is the measurement noise. The Kalman filter is an optimal estimator if the noise components are Gaussian and the transition matrices are linear. When a measurement is available, it is used as a measure of the reliability of the measurement. In addition, Kalman gain (*K*) is the key parameter for weighing the measured and predicted states. The corrected state is estimated using Equation (3). Kalman gain and the error covariance matrix (*P*) of the current state are calculated based on the errors from both current and previous steps using Equations (4) and (5), respectively:

(3) $${\hat{X}}_{t}=X_{t}^{-}+K_{t}({\tilde{Z}}_{t}-H_{t}X_{t}^{-})$$

(4) $$K_{t}=P_{t}^{-}H_{t}^{T}{\left(H_{t}P_{t}^{-}H_{t}^{T}+R_{t}\right)}^{-1}$$

(5) $$P_{t}=\frac{P_{t}^{-}H_{t}^{T}}{H_{t}P_{t}^{-}H_{t}^{T}+R_{t}}$$

A major drawback of Kalman filter is the underlying assumption of linearity and the Gaussian noise model. In its original form, the Kalman filter cannot be used for a non-linear system. Several variants of the Kalman filter, such as the extended Kalman filter (EKF) and the unscented Kalman filter, have been proposed for non-linear systems with non-Gaussian noise [14].

Sequential importance sampling (SIS) or particle filters track the posterior of a system by using a number of particles to approximate the probability distribution [15]. A particle filter has no underlying assumption regarding the linearity or the noise distribution of the system. Thus, a particle filter is applicable to a wide range of systems.

In the particle filter approach, *N* particles are sampled using the pre-known mean and variance parameters. Each particle is assigned a prior weight $W_{\text{prior}}^{i}(t)$, using a weighing function (ℱ):

(6) $$X_{t}^{i}=N\{u_{t},P_{t}\}$$

(7) $$W_{\text{prior}}^{i}(t)=F_{1}(X^{i})$$

The observation likelihood (*L_t_*) of a particle is calculated using Bayes' formula (Equation (8)):

(8) $$P(X_{t}|z_{0:t})=\int CP(X_{t}|X_{t-1},z_{0:t-1})P(z_{t}|X_{t})dx$$

Posterior weight (*W_post_*) is assigned to each particle using the prior weight and the observation likelihood using Equation (9). Subsequently, the final state *X_t_* is calculated as the weighted sum of the particles using Equation (10):

(9) $$W_{\text{post}}=\frac{W_{\text{prior}}\times L_{t}}{C}$$

(10) $$X_{t}=\sum_{i=0}^{N}W_{\text{post}}(i)*X^{i}$$

The particle filters are applicable to systems with any error model. Herein, we chose to compare the proposed approach with the gradient descent approach [12] and the autoregressive model-based Kalman filter approach [7], since these approaches and their variants are used widely for magnetic distortion compensation.

## 3. Proposed Approach

### 3.1. Overview

The Earth's ambient magnetic field and gravity vector are used as reference vectors for orientation estimation, under conditions of no distortion and quasistatic movement. During motion, the presence of magnetic distortion is detected by using magnetic strength in conjunction with the derived magnetic dip angle. The magnetic dip angle is derived by using accelerometer and magnetometer measurements. After the magnetic distortion has been detected, a particle filter is used to mitigate the magnetic distortion effects. The cost function and the variance (which controls the spread of particles in the resampling step) are adapted based on the proximity of the ferrous or magnetic object. When distortion is detected, adaptation reduces the reliance of the estimation on the magnetometer readings and places more emphasis on the results of dead reckoning based on the gyroscope readings. When the movement is in the quasistatic range, the cost function to evaluate each orientation particle is based on both the inclination estimates from accelerometers and the heading estimates from the magnetometers. Under fast acceleration, the cost function is adapted, so that it uses the heading estimated from magnetometers and the magnetic dip angle derived from the world magnetic model (WMM) to ensure isolation from corrupted inclination measurements provided by the accelerometers. The quasistatic acceleration threshold is derived prior to motion capture and is application-specific. For example, the quasistatic threshold is higher in the motion capture of sport performances and significantly lower for rehabilitation exercises.

The proposed 3D orientation estimation algorithm is presented in the following two subsections. The first subsection presents an insight into the magnetic distortion detection using both magnetic strength and magnetic dip angle. The second subsection presents the details of the particle filter for orientation estimation.

### 3.2. Magnetic Distortion Detection

#### 3.2.1. Insight

The magnetic dip angle is the angle between the lines of flux of the Earth's magnetic field and the surface of the Earth. During our experiments with magnetometers, we noted that the dip angle changes faster than the magnetic strength as the AHRS enters areas with magnetic distortions.

**Figure 2.**
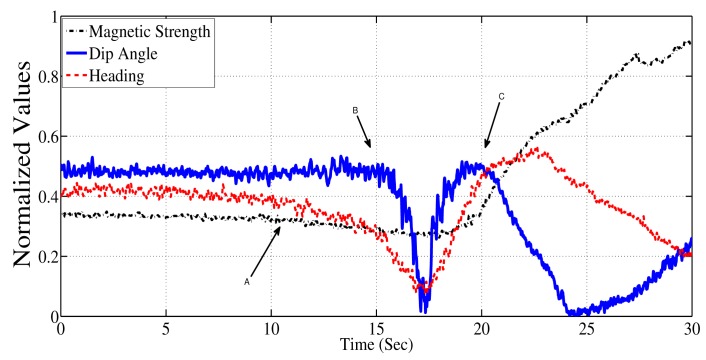
Scenario where the proposed method for detection is more applicable.

In an experiment, a three-axis magnetometer was kept static and a ferrous object was moved gradually towards the magnetometer. The movement was linear in a horizontal plane. Figure 2 shows the measurements as the ferrous object moves towards and past the magnetometer (starting from one meter north of the magnetometer and ending one meter to the south of the magnetometer). It is evident from the figure that the the heading estimate is affected first; slowly, initially, but significantly after the 12th second (Point A in the Figure 2). The dip angle is impacted second, after the 15th second (Point B). Finally, the field strength is significantly changed after the 17th second (Point C). If an algorithm were to rely solely on the change in magnetic field strength, then the presence of the disturbance would be undetected or detected late in such circumstances. Thus, it is desirable to use both magnetic dip angle and magnetic strength to detect the presence of magnetic distortions.

#### 3.2.2. Distortion Detection Method

**Figure 3.**
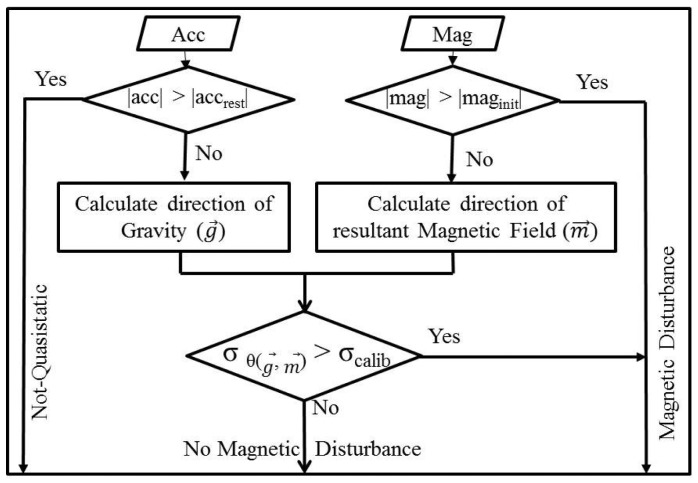
The approach used for magnetic field distortion detection.

Figure 3 illustrates the proposed algorithm for detecting magnetic distortion. Both the accelerometer and magnetometer are used to derive the magnetic dip angle. The primary check for distortion detection is comparing the current magnetic strength (|*mag*|) to a prior threshold (*mag_init_*). The threshold is calculated by placing the AHRS in a magnetically clean environment (*i.e.*, away from the magnetic/ferrous objects) for 5 s. The mean value over this period is used as the threshold. During the rest period, the floor noise of the magnetic dip angle (*σ_calib_*) is calculated.

If the primary check using magnetic strength alone detects no magnetic distortion, accelerometers are used in conjunction with the magnetometer to derive the magnetic dip angle. The quasistatic acceleration range (*acc_rest_*) is determined prior to the start of movements. The range defined as quasistatic depends on the application; examples are given in the next section. If the resultant acceleration (|*acc*|) is in the quasistatic range, the angle (*θ_dip_*) between the gravity vector and magnetic field measured by the magnetometer is derived as:

(11) $$\theta_{dip}=(pi/2)-\arccos(\vec{g}\cdot \vec{m})$$

where *g⃗* and *m⃗* are the gravity vector and Earth's magnetic field measured in the sensor frame of reference, respectively. The expected value of the dip angle at any location can be calculated using the world magnetic model (WMM) [16]. The deviation (*σ_dip_*) of the derived dip angle (*θ_dip_*) from the expected dip angle is used as a criterion for detecting magnetic distortion. If the current deviation is greater than the noise floor (*σ_calib_*), then magnetic distortion is detected.

**Figure 4.**
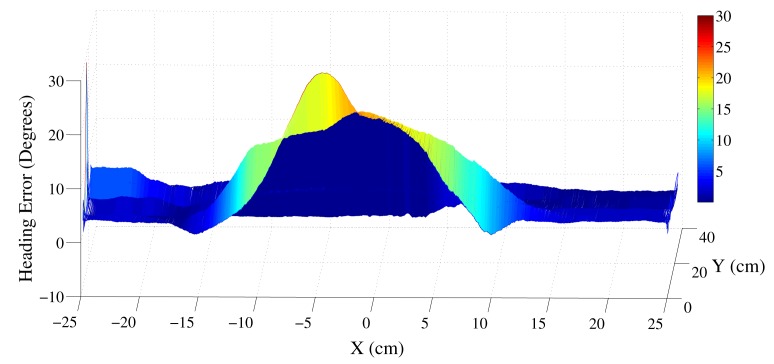
Magnitude of orientation error when the AHRS is moved in a linear trajectory across a magnetic object.

**Figure 5.**
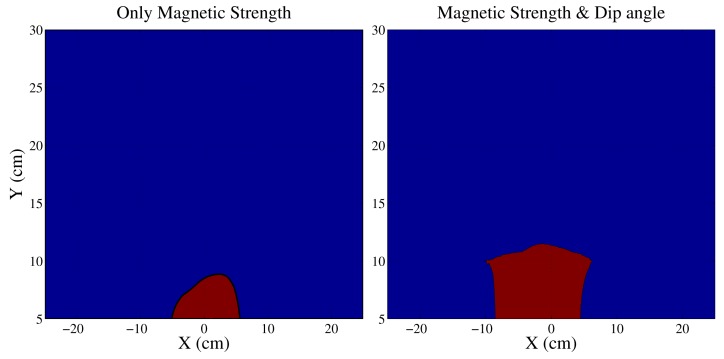
Contour plot showing the areas that are detected as magnetically distorted using magnetic field strength only (**left**) and magnetic field strength and dip angle (**right**).

The method was verified experimentally by moving an AHRS in a linear trajectory across a magnetic object. A Shimmer magnetometer, consisting of a magnetometer, accelerometer and a gyroscope, was used for experiments [17]. The magnetometer was moved across an active desktop speaker. Figure 4 illustrates the magnitudes of the heading error, when an AHRS is moved 1 m from either side of the object in the X-direction (east-west). This linear X-trajectory was repeated at distances of −1 to 1 m in the Y direction (north-south) from the material. It is evident from Figure 4 that the heading error is observed when the object is at a distance of approximately 15 cm from the speaker. The distortions are significant when the AHRS is between −10 to +15 cm separation in the X-direction from the object. The proposed detection algorithm was applied to the AHRS data with and without the magnetic dip angle information. The results are shown in Figure 5. It is evident that the detection is more sensitive when using both the magnetic strength and magnetic dip angle.

### 3.3. Motion Tracking filter

**Figure 6.**
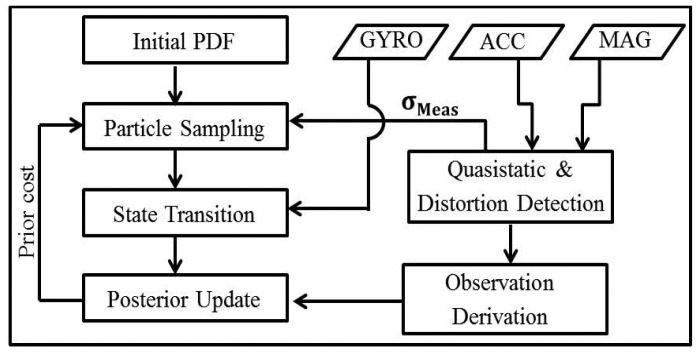
Overview of the particle filter approach used for orientation estimation.

As is conventional when tracking motion, a filter is used to improve the accuracy of orientation estimation [7,18]. When magnetic readings are not subject to magnetic distortion, filtering is performed according to the magnetometer, accelerator and gyroscope readings, as normal. However, during magnetic distortions, orientation is estimated by giving more weight to dead reckoning based on the gyroscope readings only. Kalman and particle filters are commonly used for motion tracking applications [13,15]. Herein, we use a particle filter for tracking to obviate the need for any underlying assumptions regarding the noise distributions and for motion model. The particle filter used in the proposed algorithm is shown in Figure 6. The output of the filter is a four element quaternion representation of the current 3D orientation in the Earth-fixed global frame of reference. The prior estimation of orientation is done using the standard state-transition equation using gyroscope readings as the input. The particle resampling is done based on the presence or absence of magnetic distortions. The cost function for evaluating each orientation particle is adapted based on the motion ranges and magnetic distortions. The particle resampling, likelihood calculation, posterior estimation and other components of the filter are detailed in the following subsections.

#### 3.3.1. State Vector

Quaternions were chosen to represent the state, because they do not have the problems associated with Euler angles, such as gimbal lock. For further reading on quaternions, the reader is referred to [19]. In this text, ${\hat{q}}_{i}^{-}$ represents the prior estimate of the quaternion at a time instant *i* and ${\hat{q}}_{i}^{+}$ represents the posterior estimate. The rate of change of quaternion is represented by *q̇_i_*. The state vector ( ${\hat{q}}_{i}^{-}$) consists of four elements:

(12) $$\begin{array}{ccc} {\hat{q}}_{i}^{-}=\left[\begin{array}{cccc} q_{0} & q_{1} & q_{2} & q_{3} \end{array}\right] & \text{where}, & \left\|{\hat{q}}_{i}^{-}\right\|=1 \end{array}$$

#### 3.3.2. Process Model

The angular velocity of the AHRS is measured by the gyroscope and is used as input to the state space transition calculation. The angular velocities are measured for each axis and are transformed to quaternion rates using the following equation:

(13) $$\begin{array}{ccc} {\dot{q}}_{i}=\frac{1}{2}q_{i}\left[\begin{array}{c} 0\\ \omega \end{array}\right] & \text{where}, & \omega =\left[\begin{array}{ccc} \omega_{x} & \omega_{y} & \omega_{z} \end{array}\right] \end{array}$$

The quaternion rate (*q̇_i_*) is used to predict the next state ( ${\hat{q}}_{i+1}^{-}$) using the following equation:

(14) $${\hat{q}}_{i+1}^{-}={\hat{q}}_{i}^{+}+{\dot{q}}_{i}\Delta t$$

Here, ${\hat{q}}_{i}^{-}$, ${\hat{q}}_{i}^{+}$ represent prior and posterior estimates of the state, respectively, and *t* represents the sample period. Gyroscope measurements are corrupted with floor noise and a varying offset. We model the effects of gyroscope process noise during particle resampling, as discussed in a later section.

#### 3.3.3. Measurement Model

**Figure 7.**
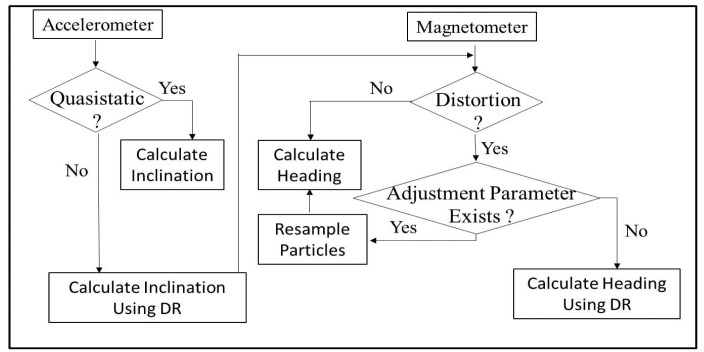
Approach for deriving heading and inclination under various scenarios.

The measurement model is adaptive, and the outputs are based on the presence or absence of magnetic distortions in the sensing environment. Figure 7 illustrates the approach used for calculating heading and inclination under various scenarios. We will look at the two scenarios separately in the following subsections.

**Magnetic Distortion Free Environment:** If no magnetic distortion is present, then the heading is calculated using the conventional method. The global frame of reference is north-east-up (X-Y-Z). The orthogonal axes of gyroscope, magnetometer and accelerometer are assumed to be aligned with each other. Yaw (*i.e.*, the rotation around the Z-axis, *ϕ*) and pitch (*i.e.*, the rotation around the Y-axis, *θ*) are calculated by solving the equation:

(15) $$R_{i}^{SG}\times \left(\begin{array}{c} A_{x}\\ A_{y}\\ A_{z} \end{array}\right)=\left(\begin{array}{c} 0\\ 0\\ -g \end{array}\right)$$

where *A_x_, A_y_* and *A_z_* are the accelerometer readings in the sensor frame of reference. $R_{i}^{SG}$ is the direction cosine matrix (DCM) of the global frame with respect to the local sensor frame of reference. Using the yaw and pitch angles, the magnetometer readings are de-rotated to align with the horizontal plane. The equivalent magnetic strengths after de-rotation are calculated as:

(16) $$\left(\begin{array}{c} M_{x}\cos\theta +M_{y}\sin\theta \sin\phi +M_{z}\sin\theta \cos\phi \\ M_{y}\cos\phi -M_{z}\sin\phi \\ -M_{x}\sin\theta +M_{y}\cos\theta \sin\phi +M_{z}\cos\theta \cos\phi \end{array}\right)$$

Roll (*ψ*) is calculated from the horizontal components of the derotated magnetic field values. The corresponding quaternion can be derived from the Euler angles using the following equation:

(17) $${\tilde{q}}_{i}=\left(\begin{array}{c} c1c2c3-s1c2s3\\ c1c3s2+s1s2s3\\ c1s2s3-s1c3s2\\ c1c2s3+c2c3s1 \end{array}\right)$$

where c1 = cos(*ϕ*/2), c2 = cos(*θ*/2), c3 = cos(*ψ*/2) and *s_j_* represent the sine of respective Euler angles.

**Magnetically Distorted Environment:** Under the presence of magnetic distortions, the heading calculated using magnetometer values cannot be directly input as a measurement to the filter. The measurement for the filter is calculated as a weighted sum of gyroscope heading estimates and magnetometer heading estimates. Dip angle is used as a criteria for weighting the current noisy measurement. The weight (*k*) is calculated as follows:

(18) $$\delta_{\theta }=\theta_{dip}-\alpha_{\text{calib}}$$

(19) $$k=\{\begin{array}{ll} 1, & \text{No distortion}\\ \frac{1}{\text{floor}(\sigma_{\delta \theta }/\sigma_{dip})}, & \text{Distortion} \end{array}$$

(20) $${\tilde{q}}_{i}=k*{\tilde{q}}_{i}+(1-k)*{\hat{q}}_{i}^{-}$$

where, *θ_dip_* is the current derived dip angle and *α_calib_* is the dip angle determined prior to the motion as explained previously. The difference between the current derived dip angle and the calibration dip angle is represented by *δ_θ_*, and the standard deviation of the derived dip angle and the difference between the derived and the calibrated dip angles are represented by *σ_δθ_* and *σ_dip_*, respectively.

**Calculating Quasistatic Thresholds:** The Earth's gravity vector is used as one of the reference vectors during orientation estimation. The gravity vector is estimated from the accelerometer readings. Accelerometers measure the vector sum of gravity vector and external accelerations. Thus, under static conditions, the accelerometers can be used to estimate the gravity vector accurately. Under conditions of external accelerations, the error in determining the gravity vector from accelerometers is proportional to the magnitude of the external acceleration. Herein, we determine the quasistatic ranges based on how much error can be tolerated in gravity vector estimation. For healthcare rehabilitation motion capture applications, an error or five degrees is acceptable [20]. The threshold for total acceleration was calculated as the maximum acceleration, which will introduce an error of five degrees when the acceleration is perpendicular to the Earth's gravity vector. Thus, we calculated the threshold for total acceleration (*i.e.*, the sum of gravity and applied acceleration) as 10.1 m/s^2^. The accelerations measured by the accelerometer below this threshold were regarded as being within the quasistatic range. When accelerations are not in the quasistatic range, a weighting approach is used described in Equations (9) and (10).

#### 3.3.4. Weighting and Resampling

When performing sampling in the state space, no underlying probability distribution function is assumed, because the system is unaware of the direction and strength of magnetic distortions. Uniform distribution is assumed when particles are sampled. For sampling, the current gyroscope reading (*ω_i_*) is used as the mean value, and the standard deviation is the floor noise of the gyroscope (*σ_ω_*).

(21) $$q_{i,j}=F({\hat{q}}_{i}^{-},N\{\omega_{i},\sigma_{\omega }\})$$

where *q_i,j_* represents the *j* – *th* particle at the *i* – *th* sample. The function ℱ calculates quaternion rates from angular velocities. For calculating likelihood, the quaternion particles are converted to direction cosine matrices (DCM).

(22) $$DCM_{\text{diff}}^{j}=DCM_{{\tilde{q}}_{i}}-DCM_{*{\hat{q}}_{i,j}}$$

The columns of difference between the DCM ( $DCM_{\text{diff}}^{j}$), give the corresponding difference between the orthogonal unit vectors (Δ*r_x_*, Δ*r_y_*, Δ*r_z_*). This difference is used as the axes of an ellipsoid and, the volume of the ellipsoid is used to calculate the likelihood (*L_j_*) parameter of the particle.

(23) $$L_{j}\propto \frac{1}{\Delta r_{x}*\Delta r_{y}*\Delta r_{z}}$$

The likelihood is used to update the posterior probability, and the weighted sum of particles is used to calculate the final estimate of the quaternion, as described in Equations (14) and (15), respectively.

(24) $$w_{\text{post}}=\frac{w_{\text{prior}}\times L_{j}}{C}$$

(25) $${\hat{q}}_{i}^{+}=\sum_{j=0}^{N}W_{\text{post}}(j)*{\hat{q}}_{i,j}^{-}$$

The posterior weight of the particle, the prior weight of the particle and the conditional probability are represented by *w_post_*, * w_prior_* and *C*, respectively.

## 4. Experimental Results and Discussion

**Figure 8.**
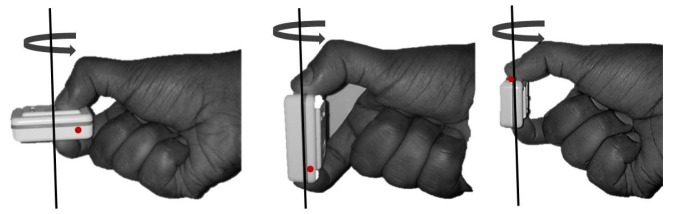
AHRS rotation used for removing hard iron offsets.

**Figure 9.**
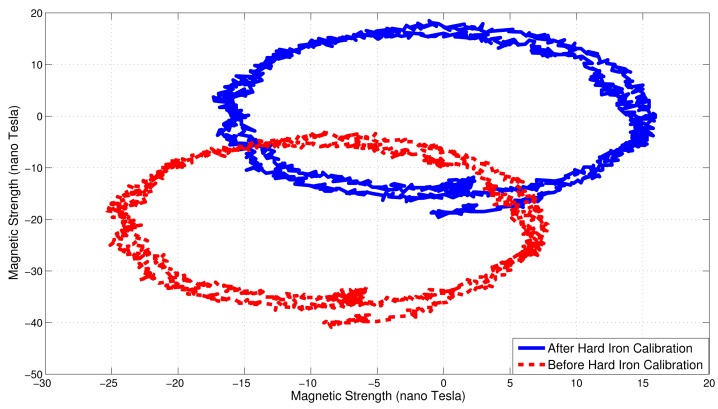
Hard iron errors are calibrated by a 360° horizontal rotation.

A nine DOF Shimmer sensor, consisting of a three-axis accelerometer, three-axis gyroscope and three-axis magnetometer, was used for experimental validation [17]. The motion capture environment was a typical indoor office space consisting of a desktop, office drawer, speakers and other materials in the vicinity. The AHRS data were sampled at 200 Hz and the data passed through a 50 Hz low pass filter. The captured data was processed in MATLAB (The MathWorks Inc., Natick, MA, USA). The Shimmer was calibrated for constant hard iron errors in the space. These constant hard iron errors are the additive magnetic fields that have a constant magnitude and direction at all times. The calibration was performed by means of a horizontal 360° rotation, as shown in Figure 8. Figure 9 shows the plot of horizontal magnetic strengths for two full rotations of the AHRS, before and after the calibration step. It is evident from the figure that the constant offset caused by the hard iron error is removed after the calibration procedure.

The proposed algorithm was tested in the static condition, in the vicinity of various ferrous objects commonly present in indoor environments. The AHRS was kept static on a horizontal surface, and three different ferrous objects were moved gradually towards the AHRS. The separation between the AHRS and ferrous object at the closest point was 5 cm. Table 1 lists the objects and the strength of the induced magnetic distortions. The RMS heading error over a 1-s period was calculated. The algorithm performed well in the presence of highly magnetic materials, such as a PC speaker, as well as in the presence of lesser magnetic materials, such as a metallic can. The errors were dependent on the angle and separation of the ferrous material and the AHRS. As shown in Table 1, the error in orientation obtained using the proposed method was less than five degrees in each case.

**Figure 10.**
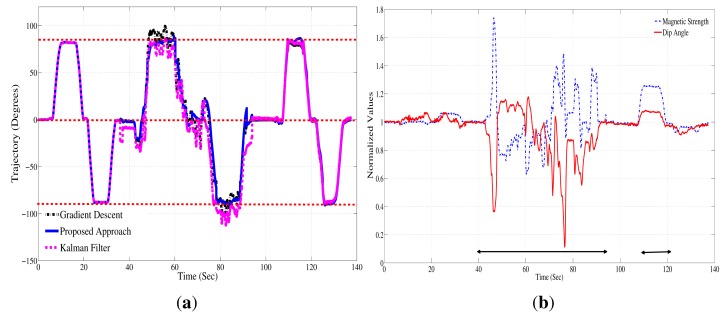
(**a**) Trajectory estimation during movement of ±90° along three axes; (**b**) normalized magnetic strength values and magnetic dip angles used for distortion detection. The arrows indicate the time period where measurements are affected by magnetic distortions.

The method was tested in dynamic experiments with a moving AHRS. The AHRS was mounted on a rotating platform that allows ±360° rotations along three orthogonal axes. The platform was rotated ±90° along all three axes. This motion was chosen, because it mirrors a basic template of rehabilitation exercises in which a patient performs repetitive flexion-extension of the arms. Figure 10a shows the results of the experiment. Magnetic distortion was introduced during the motion by placing a PC speaker close to the rotating platform between the 40th and 90th second (approximately a 10-cm distance from the speaker). The Earth's ambient magnetic field is distorted in the presence of the speaker. Figure 10b shows the normalized value for magnetic strength measured by the magnetometer. It can be seen that during magnetic distortion, the magnetic strength and dip angle change. The intervals in which distortion were introduced is depicted by arrows in Figure 10b. The result of the proposed approach was compared to the gradient descent approach proposed by Magdwick [18] and to the Kalman filter approach in [7]. Table 2 enumerates the RMS and the maximum peak-to-peak errors observed during our experiments. The maximum peak-to-peak error was less than 5° (3.4° RMS) on the trajectory estimated by the proposed approach, whereas the gradient descent approach showed a maximum error of 10° (6° RMS); the Kalman filter approach showed an error of up to 12° peak-to-peak (9.26° RMS); and the uncompensated trajectory had error of up to 23° (15.4° RMS). It should be noted that the values of the error are dependent on the type of ferrous material and the orientation of AHRS with respect to the ferrous material.

**Figure 11.**
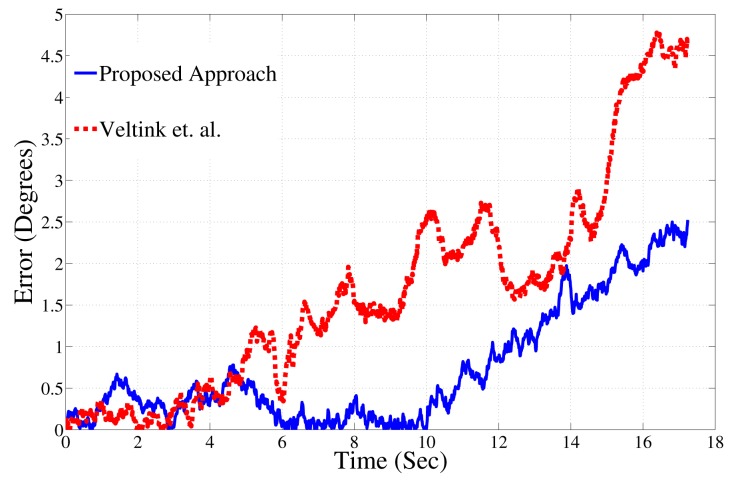
Proposed algorithm outperforms Kalman filter approach during high acceleration.

The proposed algorithm was tested under various speeds of AHRS motion. In accordance with a study that reports the range of acceleration during a sit-to-stand exercise [21], the algorithm was tested by carrying out pitch, yaw and roll rotations with accelerations ranging from zero to 3.5 *g* (where **g** = 9.8 m/s^2^). The acceleration reported here is the total magnitude of the acceleration measured by the accelerometer. Figure 11 compares the heading results for a experiment in which the AHRS was moved at 3 *g*. Due to the high acceleration, the inclination estimates were corrupted with noise. The approach used for fast accelerations is compared to a Kalman filter approach [22], which measures inclination under various ranges of accelerations of the human body. This approach was chosen for comparison, because it specifically models the accelerations using an auto-regressive process. Under conditions of fast acceleration, the proposed approach outperforms the candidate Kalman filter approach. The Kalman filter error is up to 5° in 20 s, whereas the proposed approach shows an error of 2°. Thus, the proposed approach is more robust to accelerations typically encountered during human body rehab movements.

**Figure 12.**
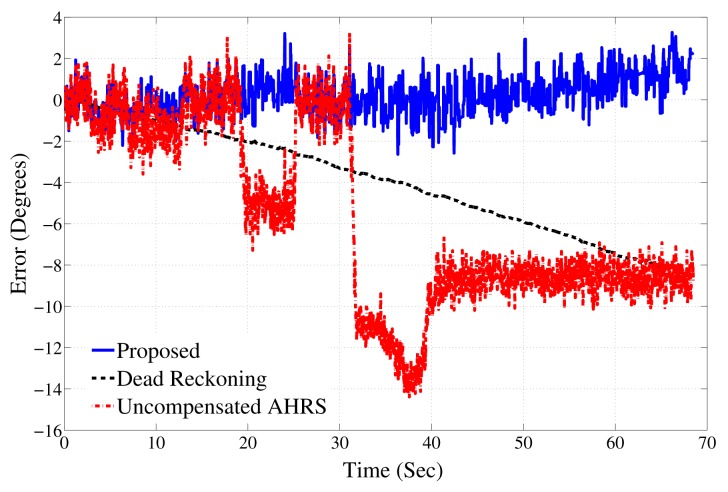
Accuracy of the proposed algorithm in the static case.

So far, we have investigated the performance of the estimation algorithm when the AHRS is moving and the ferrous material is static. In order to test the accuracy for mobile ferrous materials, the AHRS was kept stationary on a desk, and a ferrous material was placed 1 cm away from the AHRS between the 25th and 30th second. Another ferrous material was moved towards the AHRS and was kept in the vicinity for the rest of the recordings. The AHRS was static during the whole process. Figure 12 depicts the results for this experiment. The errors in orientation estimates are shown for the proposed approach, using dead reckoning and the uncompensated AHRS estimation approach. The results show up to a 14° error in the uncompensated approach. The error grows 10° per minute when only the gyroscopes are used for orientation estimation.

## 5. Conclusions and Future Work

A particle filter-based approach for the detection and mitigation of the effects of magnetic distortions on orientation estimation using an AHRS is proposed. It is shown in our experiments that the method for distortion detection is useful for both slow and fast moving objects. The improvements are up to 7° compared to the previous approach in the presence of magnetic distortions, such as in the vicinity of a speaker. The proposed method shows good performance in the presence of ferrous objects of different types, shapes and sizes. Work in the near future will focus on the possibility of differentiating between different locations and, thus, mapping the magnetic distortions, based on the pair of magnetic strength and magnetic dip angle in a magnetically distorted area.

## Figures and Tables

**Table 1.** The ferrous objects used.

**Distortion Type**	**Magnetic Flux (Gauss)**	**RMS Error (Deg) (Proposed)**	**RMS Error (Deg) (Uncompensated)**
None	0.49	0.4	0.5
Metallic Can	0.55	1.5	5.4
Portable HDD	1.15	2.8	7.2
Speaker	5.4	3	10.5

**Table 2.** Comparison of maximum peak-to-peak heading error and RMS error using various methods.

**Estimation Method**	**Peak-To-Peak Error (Deg)**	**RMS Error (Deg)**
Proposed	5	3.4
Gradient Descent	10	6
Kalman filter	12	9.26
Uncompensated	23	15.4
